# Streaming Current for Surfaces Covered by Square and Hexagonal Monolayers of Spherical Particles

**DOI:** 10.1021/acsomega.3c05603

**Published:** 2023-11-14

**Authors:** Jerzy Blawzdziewicz, Zbigniew Adamczyk, Maria L. Ekiel-Jeżewska

**Affiliations:** † Department of Mechanical Engineering, Texas Tech University, Lubbock, Texas 79409, United States; ‡ Department of Physics and Astronomy, Texas Tech University, Lubbock, Texas 79409, United States; § Jerzy Haber Institute of Catalysis and Surface Chemistry, Polish Academy of Sciences, Niezapominajek 8, Kraków 30-239, Poland; ∥ Institute of Fundamental Technological Research, Polish Academy of Sciences, Pawińskiego St. 5B, Warsaw 02-106, Poland

## Abstract

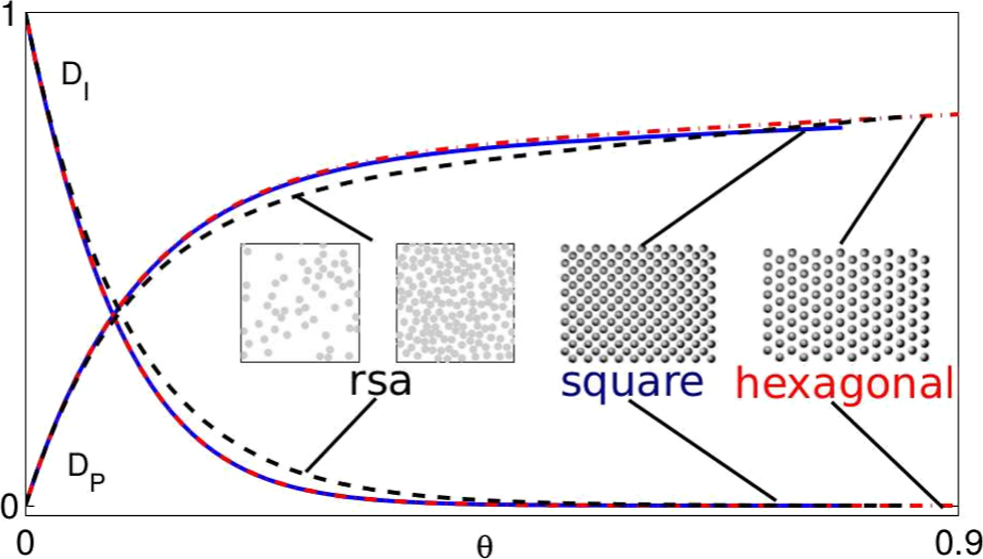

The interface and particle contributions to the streaming current of flat substrates covered with ordered square or hexagonal monolayers of spherical particles were theoretically evaluated for particle coverage up to close packing. The exact numerical results were approximated using fitting functions that contain exponential and linear terms to account for hydrodynamic screening and charge convection from the particle surfaces exposed to external flow. According to our calculations, the streaming currents for the ordered and random particle arrangements differ within a typical experimental error. Thus, streaming-current measurements, supplemented with our fitting functions, can be conveniently used to evaluate the particle coverage without detailed knowledge of the particle distribution. Our results for equal interface and particle ζ-potentials indicate that roughness can reduce the streaming current by more than 30%, even in the limit of the small size of spherical roughness asperities.

## Introduction

Deposition of nano- or microscale particles on solid surfaces is of great importance in a variety of fields of science and technology.^1−10^ Particle-covered surfaces are used, for example, as functional materials in electro-optical devices,^11−13^ biosensors,^14−17^ biomaterials,^18,19^ and plasmon-resonance spectroscopy devices.^1^ In other applications (e.g., medical devices and membrane filtration systems), particle deposition needs to be prevented to avoid surface fouling.^20^

A promising class of methods that can be used in situ to monitor deposition of nanoparticle monolayers under various physicochemical conditions consists of electrokinetic methods based on the measurements of the streaming current (or its derivative parameter, the streaming potential).^3,8^ For example, these techniques were applied to quantitatively evaluate the deposition kinetics^9,21,22^ and to determine the mechanisms of globular protein adsorption on solid/electrolyte interfaces.^23−25^

While a continuous measurement of the streaming current (or streaming potential) provides a convenient means for monitoring the deposition process, an accurate quantitative interpretation of the measurement results is not straightforward. This difficulty stems from the fact that the streaming current depends not only on the particle coverage (area fraction) θ of the particle monolayer but also on its other geometrical characteristics, such as particle shape and distribution. Thus, to fully utilize the power of the electrokinetic methods for monitoring of particle deposition, a thorough analysis of this dependence is required.

Recently, an accurate theoretical method has been developed for evaluating the streaming current produced by an arbitrary distribution of spherical particles adsorbed on a planar surface.^26,27^ This method has been used to determine the streaming current for equilibrium^26,27^ and random-sequential adsorption (RSA)^27^ distributions of the deposited particles. Accurate theoretical expressions for the streaming current as a function of the area fraction of adsorbed particles were provided^26,27^ and successfully used to interpret electrokinetic measurements under conditions where particle adsorption produces a disordered monolayer.^3,9^

**Figure 1:**
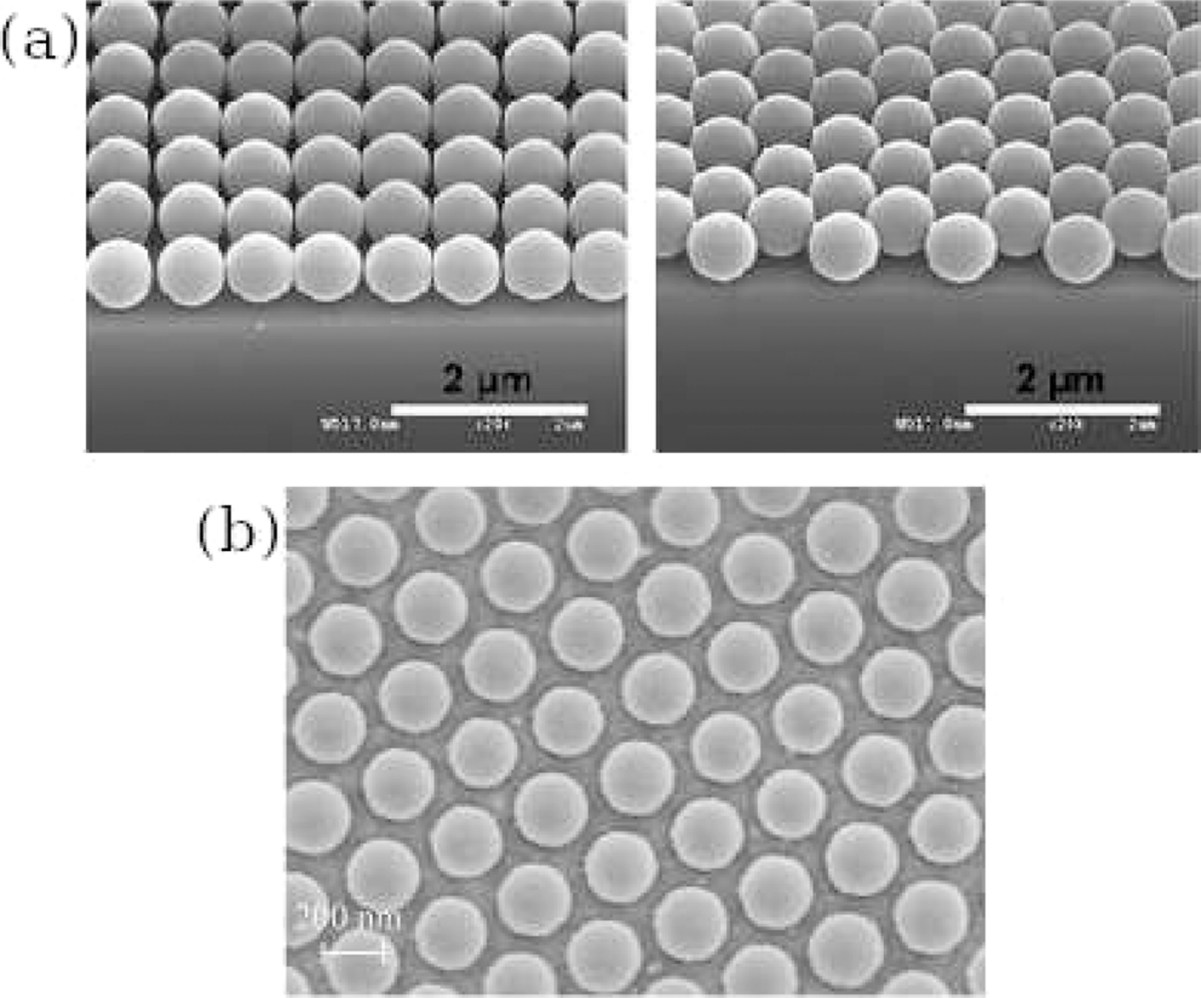
Examples of experimentally assembled 2D ordered monolayers of spherical particles. (a) SEM micrograph of a square (left) and hexagonal (right) close-packed array of silica microspheres on a Si wafer. The images are reprinted with permission from Khanh and Yoon,^29^ Copyright 2009 American Chemical Society. (b) SEM micrograph of a hexagonal monolayer of polystyrene nanospheres on the graphene substrate at an area fraction below close packing. The image reprinted with permission from Lotito and Zambelli,^30^ Copyright 2015 Elsevier.

The disordered equilibrium and RSA distributions commonly occur in adsorption processes^28^ but other distributions are also of a significant importance. In particular, ordered square and hexagonal periodic distributions of particles deposited on solid substrates (see Figure 1 reprinted from refs (29) and (30)) have been produced using a variety of experimental procedures, including the electric-field-enhanced self-assembly (electrophoresis),^31,32^ capillary-force-driven clustering,^33,34^ and the Langmuir–Blodgett assembly at liquid air interfaces followed by particle monolayer transfer to solid substrates.^35^ Such ordered structures can be used, for example, to develop band gap materials and optical filters, and therefore, they are of potentially large technological significance.

## Theoretical Methods

The main goal of the present work is to provide accurate numerical simulation data and convenient theoretical expressions for the streaming current for a planar interface covered with adsorbed spherical particles arranged on hexagonal and square ordered lattices. It is assumed that the Debye screening length λ is much smaller than the particle radius *a*_0_, i.e., λ/*a*_0_ ≪ 1. The ζ-potentials of the particles and the interface are ζ_P_ and ζ_I_, respectively. In practical applications, the ζ-potential of a planar interface can be evaluated from the uncompensated (electrokinetic) charge in the slip plane using the Gouy–Chapman formula,^36,37^ and the ζ-potential of particles can be determined by employing microelectrophoretic techniques.^9^

**Figure 2:**
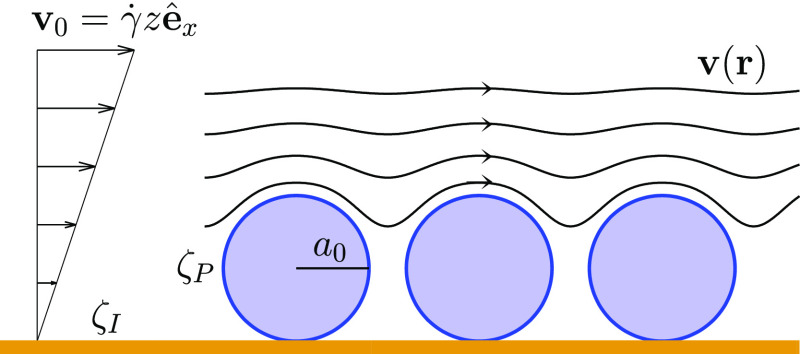
System geometry. A periodic array of spherical particles of radius *a*_0_ and ζ-potential ζ_P_ is adsorbed on a planar surface of ζ-potential ζ_I_. The particles are subject to external shear flow **v**_0_ = γ̇*z***ê**_*x*_, pointing in the *x*-direction and varying in the *z*-direction. The resulting fluid velocity field **v**(**r**) is represented by curved solid lines. For positions **r** close to the particle surface, **v**(**r**) is approximately tangential to the surface. In the regions between the particles, fluid flow is weak because of hydrodynamic screening.

The particle-covered interface (see schematic depicted in Figure 2) is subject to an external linear flow **v**_0_ = γ̇*z***ê**_*x*_ under Stokes-flow conditions. Here, *z* = 0 is the position of the interface, the fluid occupies the region *z* > 0, and **ê**_*x*_ is the unit vector along the flow direction *x*. The external linear flow is perturbed by the adsorbed particles; the resulting total flow **v**(**r**) satisfies the stick boundary conditions on the interface *S*_I_ and the particle surfaces *S*_*k*_, *k* = 1, ..., *N*, where **r** is the position vector and *N* is the number of adsorbed particles.

As described in refs (26) and (27), the flow field **v**(**r**) convects the electrokinetic charge of the Debye double layer, producing the streaming current
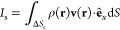
1passing through the control surface Δ*S*_c_ = *H* × , where *H* and  denote the surface dimensions in the *z* and *y* directions, respectively, and ρ(**r**) is the charge density. We note that for a square and hexagonal symmetry of the particle monolayer, the streaming current eq 1 does not depend on the flow orientation with respect to the particle lattice.

Using the Poisson equation, averaging over the control volume, integrating by parts, and splitting the resulting surface integral into the interface and particle components, eq 1 can be reduced to the expression
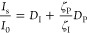
2where
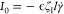
3is the streaming current, defined in eq 1 for a particle-free interface, and
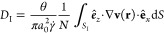
4a
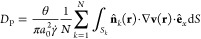
4bare the interface and particle streaming-current contributions. Here, **n̂**_***k***_ is the unit vector normal to the particle surface *S*_*k*_ (pointing into the fluid), and θ = *πa*_0_^2^*n* is the surface coverage (area fraction) of the particle monolayer, where *n* = *N*/*A* is the number of particles per unit area *A*.

As shown before,^26,27^ the interface contribution can be expressed in terms of the total hydrodynamic force  acting on the particles in the direction of the ambient flow
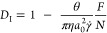
5The particle contribution
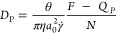
6involves the average hydrodynamic force *F* and an additional term
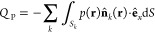
7related to the fluid pressure *p*(**r**) at the surfaces *S*_*k*_ of all the particles *k* = 1, ..., *N*.^27^

In this paper, the hydrodynamic force acting on the particles *F* and the pressure contribution were determined using the Hydromultipole numerical algorithm, based on the multipole method of solving the Stokes equations.^38,39^ The hydrodynamic wall effects were incorporated using the Cartesian representation method,^40−42^ also employed in refs (26) and (27). The calculations were performed by using 2D periodic boundary conditions in the directions parallel to the interface. There is *N* = 1 particle in a unit cell for the square lattice and *N* = 2 particles for the hexagonal lattice.

## Results and Discussion

**Figure 3:**
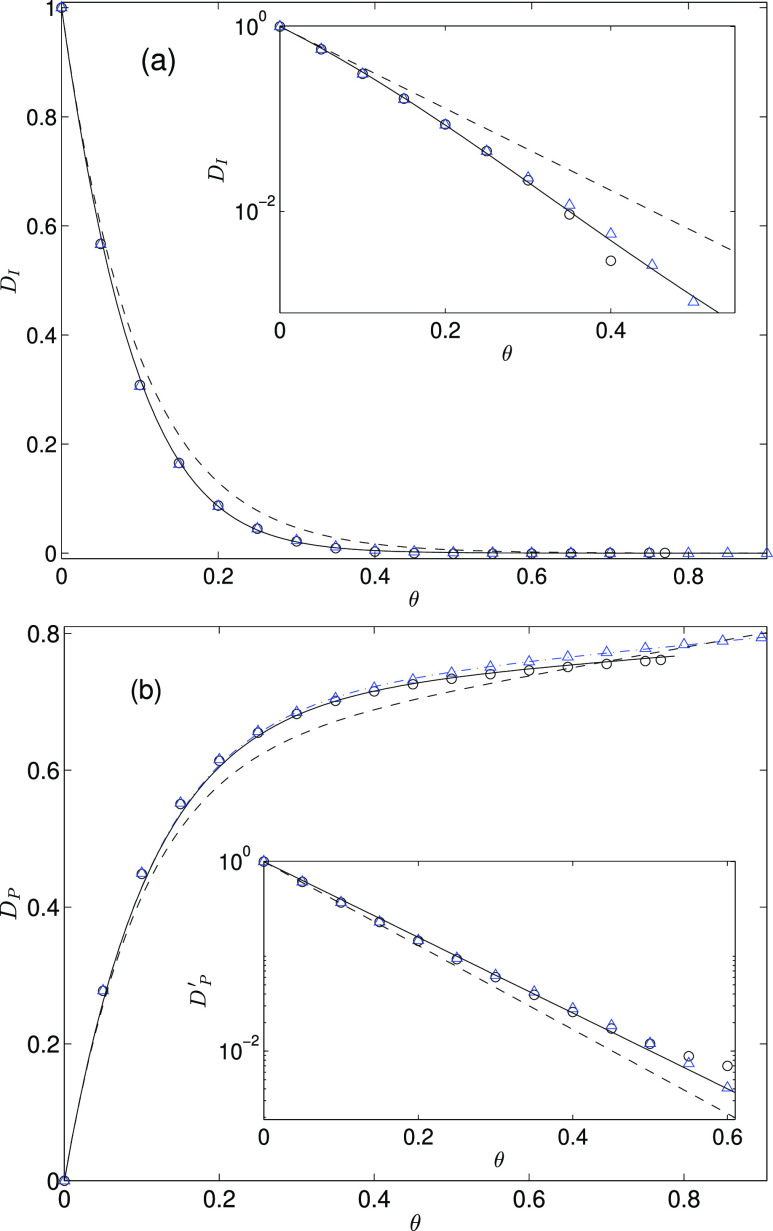
(a) Interface contribution *D*_I_ and (b) particle contribution *D*_P_ to the streaming current, shown vs the area fraction θ for a square (circles) and hexagonal (triangles) periodic lattice. (a) Solid line represents the cumulant-like approximate eq 12 for ordered particle distributions. The corresponding cumulant approximate eq 10a for random distributions is represented by the dashed line. The inset shows the data in the semilogarithmic scale. (b) Solid line represents the linear–exponential approximation given by eq 13 with the parameters listed in eqs 15 and 17 for the square particle lattice, and the dash–dot line shows approximation from eqs 13 with 16 and 17 for the hexagonal lattice. The dashed line represents the corresponding eq 10b for the random particle distributions. The inset shows the particle contribution to the streaming current with the subtracted linear part, *D*_P_^′^, (as defined by eq 18), to demonstrate the exponential approach to the linear behavior.

Our numerical results for the interface and particle contributions to the streaming current, *D*_I_ and *D*_P_, are presented in Table 1 and Figure 3 for the square and hexagonal particle configurations. The results have been obtained^27^ using a multipolar-expansion truncation order^38,39^*L* = 9 for the square lattice and *L* = 12 for the hexagonal lattice. The precision of the results is ±0.001. For each particle lattice, the data are presented for θ ≲ θ_cp_, where the close packing area fraction for the hexagonal lattice is  and for the square lattice is θ_cp_ = π/4 ≈ 0.785.

**Table 1:** Interface Contributions *A*_I_ and *D*_I_ and Particle Contributions *A*_P_ and *D*_P_ to the Streaming Current *I*_s_ for Square and Hexagonal Particle Monolayers^a^

	square				hexagonal			
θ	*A*_I_^sq^	*A*_P_^sq^	*D*_I_^sq^	*D*_P_^sq^	*A*_I_^hex^	*A*_P_^hex^	*D*_I_^hex^	*D*_P_^hex^
0	10.20371	6.50975	1.000	0.000	10.20371	6.50975	1.000	0.000
0.05	8.662	5.548	0.567	0.277	8.679	5.557	0.566	0.278
0.10	6.918	4.485	0.308	0.448	6.939	4.496	0.306	0.450
0.15	5.564	3.671	0.165	0.551	5.580	3.680	0.163	0.552
0.20	4.563	3.070	0.087	0.614	4.570	3.077	0.086	0.615
0.25	3.821	2.620	0.045	0.655	3.821	2.627	0.045	0.657
0.30	3.261	2.274	0.022	0.682	3.256	2.283	0.023	0.685
0.35	2.831	2.004	0.009	0.701	2.824	2.014	0.012	0.705
0.40	2.493	1.788	0.003	0.715	2.486	1.800	0.006	0.720
0.45	2.222	1.612	0.000	0.726	2.216	1.627	0.003	0.732
0.50	2.002	1.467	–0.001	0.734	1.998	1.484	0.001	0.742
0.55	1.820	1.346	–0.001	0.740	1.818	1.365	0.000	0.751
0.60	1.668	1.243	–0.001	0.746	1.667	1.264	0.000	0.759
0.65	1.539	1.155	0.000	0.751	1.539	1.178	0.000	0.766
0.70	1.428	1.079	0.000	0.755	1.429	1.103	0.000	0.772
0.75	1.333	1.013	0.000	0.760	1.334	1.037	0.000	0.778
0.77	1.298	0.989	0.001	0.761				
0.80					1.250	0.980	0.000	0.784
0.85					1.177	0.928	0.000	0.789
0.90					1.111	0.882	0.000	0.794

a The results for θ = 0 were obtained using the cluster-expansion method,^26^ and the remaining data were evaluated from simulations of the electrokinetic flow in a square or hexagonal unit cell.

In Table 1, we also provide the data for the associated functions
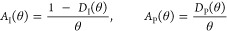
8

In the low-area-fraction limit, θ → 0, the functions eq 8 tend to the leading-order virial expansion coefficients, *D*_I_^0^ and *D*_P_^0^, in the area-fraction expansion of *D*_I_ and *D*_P_
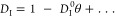
9a
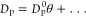
9b

The first virial coefficients *D*_I_^0^ = *A*_I_(0) and *D*_P_^0^ = *A*_P_(0) do not depend on the particle distribution. Their values, evaluated in ref (26), using the cluster expansion method, are listed in the first row of Table 1.

Our numerical data presented in Table 1 and the plots of *D*_I_ and *D*_P_ depicted in Figure 3 (open circles for the square and open triangles for the hexagonal particle lattice) show that for both ordered particle arrangements, the results are nearly identical. The data for the interface contribution are nearly the same (the differences are close to the calculation error), and the results for the particle contributions differ by less than 0.02, with the largest differences occurring at high area fractions. A similar behavior was found in numerical simulations of equilibrium and RSA arrangements of the adsorbed particles, i.e., the streaming current contributions for these random distributions are nearly indistinguishable (see the data in Table 1 and Figure 4 of ref (27)).

**Figure 4:**
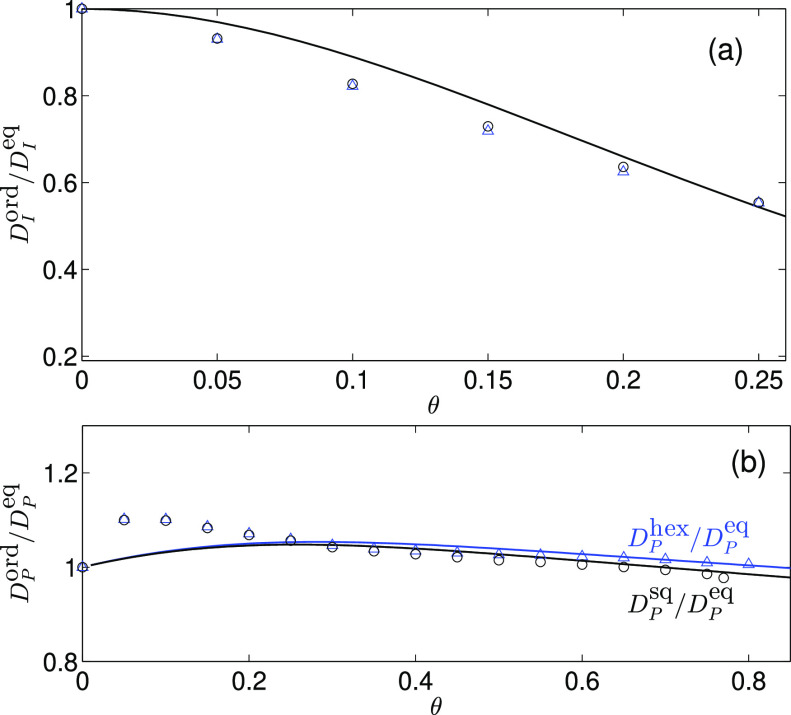
Comparison between the streaming current contributions for the ordered and random particle distributions. (a) Ratio *D*_I_^ord^/*D*_I_^eq^ between the interface streaming-current contribution for the hexagonal particle lattice, *D*_I_^ord^ = *D*_I_^hex^, (triangles) or square particle lattice, *D*_I_^ord^ = *D*_I_^sq^, (circles) and the equilibrium contribution *D*_I_^eq^. The solid line shows the ratio between the corresponding phenomenological approximations given by eqs 12, 10a and 10b. (b) Same as panel (a), except that the results are plotted for the particle distribution *D*_P_. The phenomenological expressions in this case are given by eq 10b for the equilibrium distribution and by eq 13 for the hexagonal and square distributions with the corresponding values from eqs 16 and 15 of parameters *a* and *b*.

To facilitate a comparison of our present results for particles placed on a periodic lattice with the earlier calculations for random particle distributions, Figure 3 shows our numerical data for square and hexagonal lattices (symbols) along with the cumulant approximation
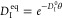
10aand linear–cumulant approximation
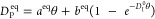
10bfor the interface and particle contributions to the streaming current for the random distributions^27^ (dashed lines). The coefficients
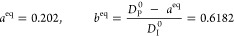
11in the above expression have been obtained by fitting eq 10b to the numerical data, and *D*_I_^0^ and *D*_P_^0^ are the first virial coefficients (which are independent of the particle distribution). Both approximations given by eqs 10a and 10b are consistent with the first-order virial expansion from eqs 9a and 9b.

We will now discuss the streaming current for the ordered particle distributions. First we focus on the behavior of the interface contribution to the streaming current *D*_I_. A comparison between our present numerical data for the ordered lattices with the cumulant approximation for the equilibrium/RSA particle distributions indicates that for both systems, the function *D*_I_(θ) decays exponentially with the area fraction. As explained in ref (27), the rapid decay of *D*_I_ stems from hydrodynamic screening of the flow near the interface by the adsorbed particles.

The initial decay rate is the same for the ordered and random cases because the first virial coefficient in expansion given by eq 9a does not depend on the particle distribution. However, for larger values of θ, the decay of *D*_I_ for ordered particle arrangements is up to 35% faster than that for the random distribution (the inset is shown in Figure 3a). This behavior implies that the hydrodynamic screening is more efficient for ordered than random distributions. The weaker screening by the random distributions (equilibrium and RSA) likely stems from the fact that in the random systems, the empty areas between particles are polydisperse.

In order to account for the variation of the decay rate of *D*_I_ with θ for the ordered distributions, we propose a simple approximation
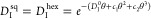
12represented by the solid line in Figure 3a. The previously evaluated^26^ leading-order virial coefficient *D*_I_^0^ = *A*_I_(0) is listed in Table 1, and the parameters *c*_1_ = −*c*_2_ = 13 have been obtained from a fit to the numerical data. The expression in eq 12 has a form of the *O*(θ^3^) cumulant expansion and is based on the approximately linear dependence of log *D*_I_ on θ (see the inset of Figure 3a), which results from hydrodynamic screening of the flow field near the interface. The corrections to the linear behavior are accounted for by the square and cubic terms. For low area fractions θ < 0.2, the absolute accuracy of the approximation given in eq 12 is better than ±0.02, for higher area fractions θ ≥ 0.2 (where *D*_I_ has already significantly decayed), the absolute precision is better than ±0.003.

In contrast to the interface contribution *D*_I_, particle contribution *D*_P_ monotonically grows with the increasing area fraction (Figure 3b). The initial growth rate is large, consistent with the value *D*_P_^0^ = 6.510 of the virial-expansion coefficient. At higher area fractions, however, the growth rate is much lower because only particle areas exposed to external flow contribute to the streaming current.

This behavior is well reflected by a combination of a linear and an exponential function
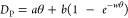
13where *a* and *w* are fitting parameters and
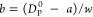
14for consistency with the virial expansion in eq 9b. By matching the expression in eq 13 to our simulation data, we find
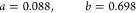
15for the square lattice and
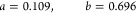
16for the hexagonal lattice, with
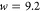
17for both the square and hexagonal particle arrangements. The fitting relation in eq 13 is analogous to the expression from eq 10b for the random particle distribution,^27^ but both the linear slope *a* and the exponential decay rate *w* are different. The absolute precision of the approximation given by eq 13 with the parameter values listed in eqs 15–17 is better than ±0.02 for θ < 0.25 and better than ±0.005 for θ ≥ 0.25, for both the hexagonal and square lattices.

The exponential approach to the linear behavior, as described by eq 13, is depicted in the inset of Figure 3*b*, where we plot the function
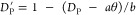
18(i.e., *D*_P_ with the subtracted linear term) for the square and hexagonal particle distributions along with the exponential fitting function *D*_P_^′^ = *e*^–*wθ*^ (solid line) and the corresponding approximation  for the random distribution (dashed line). The results show that the approach of *D*_P_ to the linear behavior is slower for the ordered distributions compared to the random distributions, but for square and hexagonal lattices, the exponent is the same.

According to the data shown in Figure 3, the absolute difference between the streaming-current contributions for the periodic and random particle arrangements is small (less than 0.065 and 0.045 for *D*_I_ and *D*_P_, respectively, which remains within the usual experimental accuracy). The largest difference occurs in the domain of moderate particle coverage, 0.1 ≲θ≲ 0.2. While the relative difference between the periodic and random results for *D*_I_ can be quite large outside the low-area-fraction region (Figure 4), this behavior occurs only when *D*_I_ is strongly reduced as a result of hydrodynamic screening. Therefore, in most cases, this difference does not have experimental significance.

**Figure 5:**
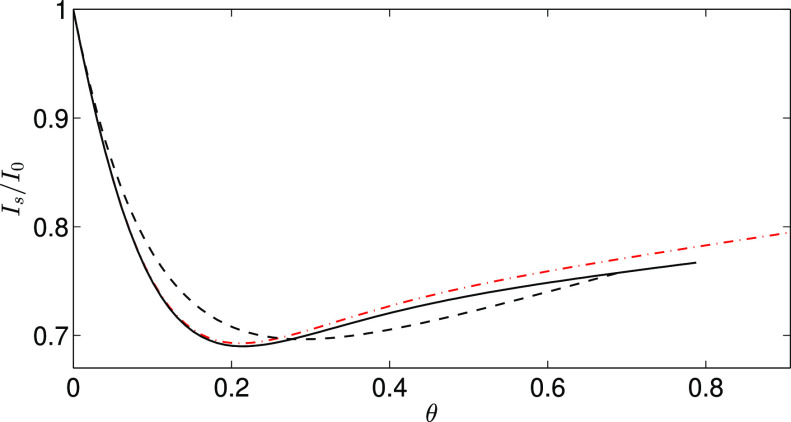
Reduction of the streaming current *I*_s_ for a rough surface with spherical asperities relative to the corresponding result *I*_0_ for a smooth surface with the same ζ-potential. The normalized streaming current *I*_s_/*I*_0_ is plotted vs the area fraction θ for the square (solid black line), hexagonal (dashed-dotted red line), and equilibrium/RSA (dashed black line) asperity arrangements.

The numerical results presented here support our earlier conclusion^27^ that surface roughness can significantly reduce the streaming current as long as the Debye length λ is much smaller than the radius of the roughness asperities *a*_0_. Such a rough surface can be modeled as a smooth interface with an attached infinite array of spherical roughness asperities with the same ζ-potential as the underlying interface. Our results for ordered particle monolayers, depicted in Figure 5, show that the streaming current is significantly smaller for a rough surface (up to about 30%) than that for a smooth surface made from the same material. This result generalizes our earlier finding for equilibrium and RSA distributions.^27^ We observe that the streaming-current reduction is relatively insensitive to the asperity arrangement, with differences smaller than 5% between *I*_s_/*I*_0_ for the square, hexagonal, and random distributions.

The streaming-current reduction by roughness is a general, universal phenomenon and is important for a correct interpretation of experiments.^27^ Since our results do not depend on the particle diameter, this reduction does not vanish when the size of roughness asperities is decreased (as long as the condition λ ≪ *a*_0_ is satisfied). Based on the above observations, we hypothesize that a similar mechanism may decrease the electrokinetic mobility of rough particles.

In conclusion, we have evaluated the interface and particle contributions, *D*_I_ and *D*_P_, to the streaming current for interfaces covered by hexagonal and square lattices formed by monolayers of spherical particles. To this end, we have used the accurate methods^38,39,41^ based on the multipole expansion of the Stokes equations to evaluate the charge convected by the flow.

We have shown that for both hexagonal and square particle arrangements, the interface contribution *D*_I_ can be approximated by a single exponential function with the *O*(θ^3^) exponent. This function is the same for both lattices, and it decays to zero at large area fractions. The particle contribution *D*_P_ can be described by a combination of a linearly increasing term and an exponentially decaying function.

The exponential decay of *D*_I_ and the exponentially decaying contribution in *D*_p_ stem from the hydrodynamic screening by the particle monolayer. The screening results in a significant reduction of the flow under the particles and the gaps between particles. We find that the screening is more effective for the periodic (square and hexagonal) than random (equilibrium and RSA) distributions. The weaker screening by the random distributions is likely due to the fact that in the random systems, the empty spaces between particles are polydisperse, and in the larger spaces, the screening is weaker, impeding the overall screening effect.

The linear behavior of *D*_P_ at large area fractions is associated with charge convection along the portions of particle surface areas that are exposed to the external flow. The total exposed area is proportional to the number of the adsorbed particles, leading to the linear increase of the streaming current in dense systems (i.e., systems where the flow near the unexposed portions of particle surfaces are already screened out).

Our numerical results and the fitting functions describing *D*_P_, shown in eqs 10b and 13, indicate that the slope *a* of the linear function to which *D*_P_ tends at large area fractions is the largest for the random distributions and the smallest for the square lattice. This dependence may be related to a different effective particle area exposed to the flow, which is influenced by the distribution of the particle neighbors.

In general, we have found that the differences in *D*_I_ and *D*_P_ between the square and hexagonal lattices are small. The differences between the ordered and random distributions are larger but still not significant. As a result, the normalized streaming current *I*_s_/*I*_0_ is almost insensitive to the specific form of the particle distribution in the adsorbed monolayer.

This is an important finding because it shows that streaming-current measurements can be reliably used to monitor the particle area fraction in a particle monolayer adsorbed on a flat surface. The above conclusion is valid for spherical particles provided that the particle distribution does not involve large density fluctuations (e.g., formation of dense clusters separated by empty or low-density regions). If there are no such fluctuations, detailed information about the particle arrangement is not necessary for the interpretation of the streaming-current measurement results. The usefulness of the streaming-current-based surface-coverage monitoring method is enhanced by the fact that our theoretical findings can be summarized by using simple phenomenological expressions to facilitate the analysis of experimental data.
